# Tumor heterogeneity: preclinical models, emerging technologies, and future applications

**DOI:** 10.3389/fonc.2023.1164535

**Published:** 2023-04-28

**Authors:** Marco Proietto, Martina Crippa, Chiara Damiani, Valentina Pasquale, Elena Sacco, Marco Vanoni, Mara Gilardi

**Affiliations:** ^1^ Next Generation Sequencing Core, The Salk Institute for Biological Studies, La Jolla, CA, United States; ^2^ Gene Expression Laboratory, The Salk Institute for Biological Studies, La Jolla, CA, United States; ^3^ NOMIS Center for Immunobiology and Microbial Pathogenesis, The Salk Institute for Biological Studies, La Jolla, CA, United States; ^4^ Vita-Salute San Raffaele University, Milan, Italy; ^5^ Experimental Imaging Center, Istituti di Ricovero e Cura a Carattere Scientifico (IRCCS) Ospedale San Raffaele, Milan, Italy; ^6^ Infrastructure Systems Biology Europe /Centre of Systems Biology (ISBE/SYSBIO) Centre of Systems Biology, Milan, Italy; ^7^ Department of Biotechnology and Biosciences, School of Sciences, University of Milano-Bicocca, Milan, Italy; ^8^ Salk Cancer Center, The Salk Institute for Biological Studies, La Jolla, CA, United States

**Keywords:** tumor microenvironment (TME), tumor heterogeneity, tumor immune microenvironment, heterogeneity models, human *in vitro* models

## Abstract

Heterogeneity describes the differences among cancer cells within and between tumors. It refers to cancer cells describing variations in morphology, transcriptional profiles, metabolism, and metastatic potential. More recently, the field has included the characterization of the tumor immune microenvironment and the depiction of the dynamics underlying the cellular interactions promoting the tumor ecosystem evolution. Heterogeneity has been found in most tumors representing one of the most challenging behaviors in cancer ecosystems. As one of the critical factors impairing the long-term efficacy of solid tumor therapy, heterogeneity leads to tumor resistance, more aggressive metastasizing, and recurrence. We review the role of the main models and the emerging single-cell and spatial genomic technologies in our understanding of tumor heterogeneity, its contribution to lethal cancer outcomes, and the physiological challenges to consider in designing cancer therapies. We highlight how tumor cells dynamically evolve because of the interactions within the tumor immune microenvironment and how to leverage this to unleash immune recognition through immunotherapy. A multidisciplinary approach grounded in novel bioinformatic and computational tools will allow reaching the integrated, multilayered knowledge of tumor heterogeneity required to implement personalized, more efficient therapies urgently required for cancer patients.

## 1 Tumor heterogeneity: a multifaceted phenomenon

The NCI Dictionary of Cancer describes cancer heterogeneity as “the differences between tumors of the same type in different patients, the differences between cancer cells within a single tumor, or the differences between a primary (original) tumor and a secondary tumor” ((NCI), n.d.). Tumor heterogeneity first originates from the clonal expansion of individually mutated cells that, interacting with the evolution of the tumor microenvironment, provide the genetic and epigenetic material upon which Darwinian and non-Darwinian evolutionary trajectories drive cancer evolution (1). The cancer phenotypic properties are modulated at the epigenetic, transcriptional, protein, and environmental levels, where different cell types contribute to the heterogeneity of the cancer tissue in both time (as the tumor evolves) and space, where the evolving composition of the tumor microenvironment—that includes dynamically interacting cancer, immune, or stromal cells—originates the ability of the cancer tissue to respond to environmental cues and access nutrients, growth factors, and oxygen. In turn, this molecular and cellular heterogeneity translates to intra- and interpatient spatiotemporal variability in the global properties of the tumor, deeply affecting drug response and disease outcome (2).

Intertumoral heterogeneity describes the tumor-by-tumor differences among different patients and is dependent on environmental factors impacting patients’ phenotypes.

**Figure 1:**
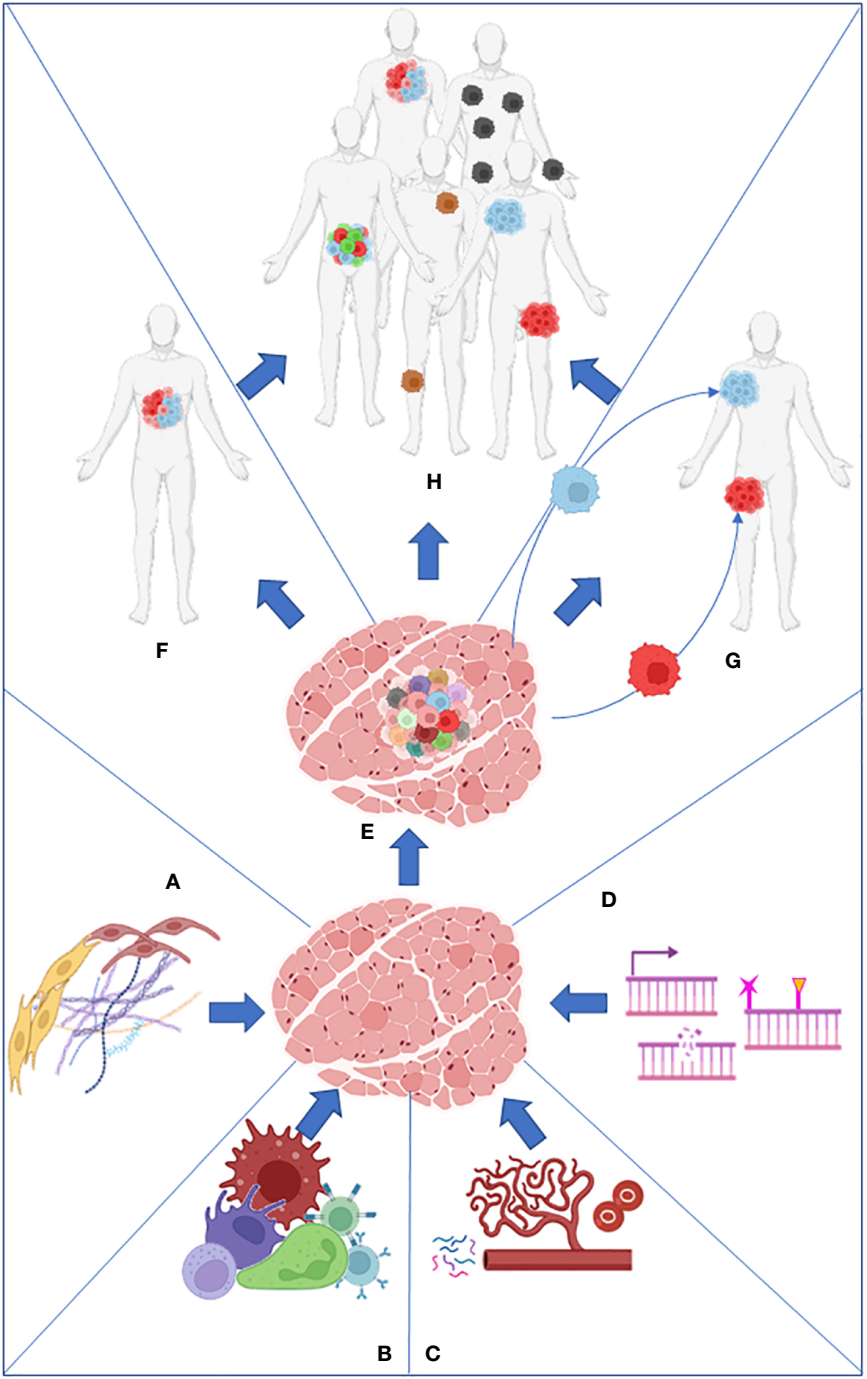
Stroma **(A)**, immune cells **(B)**, nutrients present in the microenvironment **(C)**, and intrinsic factors such as DNA damage and epigenome **(D)** work together to produce the primary tumor heterogeneity **(E)**. Intratumor heterogeneity can arise from the primary tumor **(F)** or from its metastasis **(G)**. Both processes collaborate in the establishment of intertumoral heterogeneity in the population **(H)**.

On the other hand, intratumor heterogeneity (ITH) is the cellular diversity within the same tumor or between primary and metastatic lesions. It includes copy number variations (3), epigenetic alterations, coding and non-coding somatic alterations, and transcriptome, proteome, and metabolome differences (4) (**Figure 1**).

In tumor sites, the accumulation of genetic and epigenetic alterations and chromosomal aberrations is strongly accelerated for various reasons. It can be fostered by: i) the expression of oncogenes or the loss of tumor suppressor genes that compromise the DNA repair mechanisms or the spindle assembly checkpoint, causing genomic instability; ii) exposure to endogenous or exogenous toxic factors including therapeutic agents; iii) the tumor microenvironment (TME) (nutrient limitation/hypoxia/immune system); iv) other genetic and non-genetic factors.

Among the several genes whose deregulation contributes to tumor heterogeneity, the tumor suppressor *TP53* stands out. The loss of function of the *TP53* gene determines the deregulation of the cell cycle arrest checkpoint allowing cell proliferation despite the presence of stress signals and DNA damages or skipping apoptosis also in the presence of severe DNA damages (5). Also, the deregulation of genes involved in the DNA repair system (mismatch repair or proofreading machinery) drives genome instability and subclonal heterogeneity in the tumor sites (6, 7). Large-scale chromosomal alteration events causing the loss of genetic material in the order of hundreds of genes greatly accelerate subclonal evolution and increase the tumor mutational burden (the total number of mutations per coding area of a tumor genome) which can positively or negatively correlate with prognosis and pharmacological response (8).

Exogenous factors contributing to tumor heterogeneity include physical factors, such as ionizing and non-ionizing electromagnetic radiation (UV); chemical factors such as heavy metals and toxic chemicals including drugs used in anticancer treatments; and biological factors, such as viruses, bacteria, and reactive oxygen species (ROS) arising as a by-product of the mitochondrial energy metabolism (9). In this context, it has been shown that some types of tumors such as melanoma and lung cancer have a high clonal “homogeneous coding” mutational burden due to the continuous exposure of stem cell niches to carcinogens, such as UV for skin and tobacco for lungs.

Nutrient shortage and hypoxia experienced by the cells within the core of a newly formed and non-vascularized tumor mass impose profound metabolic rearrangements, selecting clones preferring fermentative anaerobic metabolism (10–12). The TME can also affect tumor heterogeneity in terms of quantity and phenotypic characteristics of immune and stromal cells recruited at the tumor site (13–15).

The stroma and immune system’s role in tumor heterogeneity will be extensively described in Section 6.

In this manuscript, we will review established and emerging models used to study tumor heterogeneity and how the integrated use of these models and technologies can improve our knowledge of tumor heterogeneity, with a special focus on the increasing role of immune cells.

## 2 Non-human models to study heterogeneity

**Figure 2:**
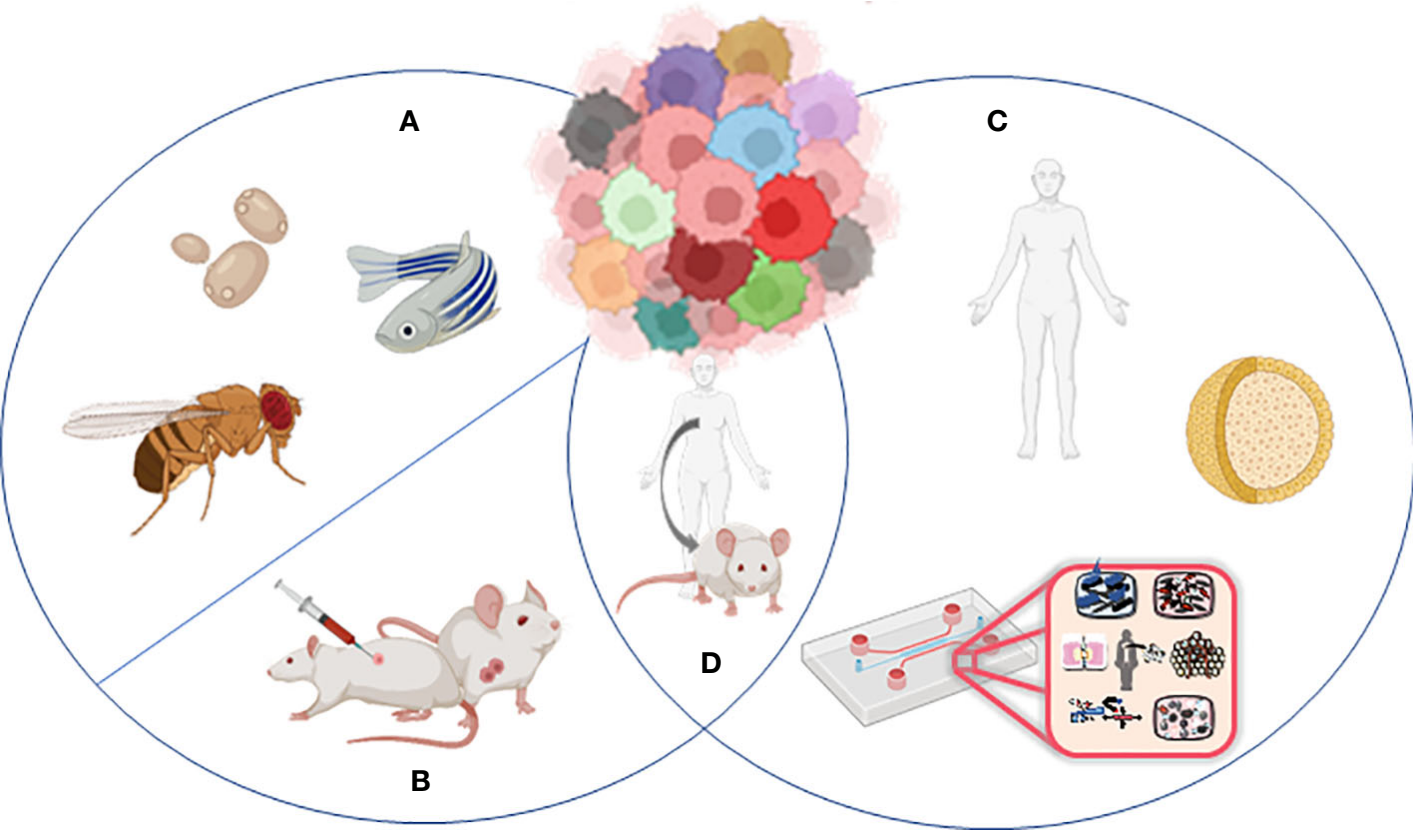
Models to study tumor heterogeneity: **(A)** non-murine models (*Drosophila melanogaster, Danio renio*, *Saccharomyces cerevisiae*), **(B)** murine models (syngeneic models, GEMMs), **(C)** human *in vitro* models (organoids, organ-on-chip), and **(D)** humanized murine models.

Animal models and cellular *in vitro* models have long been exploited to better understand the complex biological processes characterizing normal human physiology and disease (**Figure 2**).

In particular, the mouse is the most used animal model for biomedical research, discovery, and validation. More recently, novel approaches leveraging bioengineering and complex culture methods have become more present in the field. Since TME is such a complex and dynamic microenvironment, different models and more comprehensive ways to dissect the mechanism underlying heterogeneity and response to treatment have been developed. Remarkably, for every single research application and biological question, there is a right model and strategy to apply.

### 2.1 Murine models used to uncover tumor heterogeneity

The most used animal models for cancer research are genetically engineered mice (GEMMs). GEMMs are immunocompetent transgenic mice that spontaneously develop malignancies (16). GEMMs allowed the fundamental discovery that tumor development is driven by the gene loss of a tumor suppressor gene and/or an oncogene overexpression (17, 18).

While the field has relied extensively in the past 20 years on Cre/Lox models, the more recent development of CRISPR/Cas9 approaches has further accelerated the development of mouse models of human cancers.

Because the mice used in these experiments live in controlled environments and are genetically similar, tumor development and associated phenotypes are highly reproducible, allowing longitudinal studies that are more difficult in humans. Nevertheless, even in the most controlled environment, mouse tumors arising from defined genetic events do evolve to be genetically different and unique, like human tumors (19). However, mouse tumors may evolve with a lower level of genetic heterogeneity due to the absence of environmental mutagens in most cases leading to limited translational value.

A great strategy to study heterogeneity in murine models is lineage tracing allowing for the definition of the mode of tumor growth by clonal analysis. This technique has been extensively used in differentiation studies, and it has also been exploited in cancer. For example, leveraging this, Schepers et al. identified Lgr5+ stem cell activity in mouse intestinal adenomas (20).

Another simple way to mimic the human situation is to treat mice with the same carcinogens that are known to cause cancer in humans (21). For example, the 4NQO carcinogen present in tobacco has been used to induce head and neck cancer development in mice mimicking up to 94% tobacco mutational signatures (22). The importance of these types of models is supported by their extremely extensive use (23–25).

The use of GEMMs is limited by intrinsic problems, including reduced viability if the mutations occur in the germline, early death in case of simultaneous development of multiple tumors, and non-synchronous tumor development in different mice due to incomplete mutation penetrance (26). Some of these limitations can be overcome with the application of novel technologies (17, 27, 28), but mostly reliable heterogeneity studies rely on patient-derived xenograft (PDX) models (29, 30). Human tumor cells are transplanted into an immune-deficient mouse to obtain a PDX, maintaining the heterogeneity of the primary tumor (31). A greater success rate can be achieved with major immunosuppression in the animal host, and the exploitation of mice lacking B and T lymphocytes and NK cells shows a better success rate (32). An advantage of using PDXs is that aggressive tumors such as colorectal and gastric cancer have more possibilities to engraft in the host, making PDX an extremely valuable resource to study aggressive pathologies. Human hormonal treatment of transplanted mice can improve the engraftment rate of hormone-driven tumors (30).

The biological differences between mice and humans are limiting factors in the direct translation of many discoveries to the clinical setting. Thus, a growing need for a mouse model that better recapitulates the important features of human biology and immunity became more urgent. With the advent of immunotherapeutic drugs leveraging the immune system and since the mouse immune system does not always replicate the human one, new humanized mouse models providing the immune components required to test new therapies have been generated. These humanized models represent a tremendous advantage in providing a platform resembling the human response. Humanized mice with a partial or fully reconstituted immune system have been developed through stem cell transplant, and they are a promising platform to assess the efficacy of immune checkpoint inhibitors. However, since the human immune system engraftment process necessitates multiple donors, this generates a high variability in the results requiring both an increased number of donors and mice. As an alternative, knock-in mice have been developed. These models express human genes such as immune checkpoint inhibitors, PD-1, allowing efficacy studies in immune-competent systems (33).

### 2.2 Non-murine models exploited in the understanding of tumor heterogeneity

Although most of the research that focused on cancer heterogeneity has relied on primary patient samples, murine xenograft, and murine models, there are more evolutionarily distant model organisms that are genetically, histologically, and behaviorally similar to the human cancer disease, and they can have a potential key role in our understanding of cancer heterogeneity.

#### 2.2.1 Yeast

The yeast *Saccharomyces cerevisiae*—a eukaryotic unicellular organism that has long been successfully used as a model organism for human biology (34)—can grow both in liquid and solid media using different sugars to support its growth. Approximately half of the yeast genes exhibit periodic expression patterns when grown under continuous, nutrient-limited conditions. The cell cycle stage significantly exacerbates the natural variability present in the population (35). Similarly, tumor cells could respond differently to the TME according to their cell cycle stage.

Individual yeast cells respond differently to sugar sources, and variability in the expression of sugar-metabolizing genes is observed. For example, single-cell sequencing data showed that only 1.5% of cells express genes required for galactose metabolism without this sugar (36).

The group of Teusink showed that metabolic heterogeneity within a yeast population could be established and maintained without any genetic difference (37).

This metabolic variability is observable and even amplified in yeast colonies growing on solid media. The cells within a yeast colony are all the progeny from a single founder and share the same genome. Nevertheless, individual cells in a colony have different access to resources. Cells in the colony’s lower part—i.e., closer to the solid medium—have easier access to nutritional resources, expressing genes related to respiratory metabolism, while in the upper part, the cells which cannot directly access the nutrients rely on fermentation (38). As a result, in a single colony, a small number of cells survive using gluconeogenesis, releasing metabolites consumed by another subpopulation with a different metabolic phenotype (39).

#### 2.2.2 Zebrafish

Zebrafish and humans share 70% of protein-coding genes (40), and their cancers are genetically and histologically similar (41), also sharing some important drivers in the onset (42). In the field of heterogeneity studies, zebrafish optical clarity has been combined with tumor labeling and new imaging techniques by White et al., who transplanted single-cell tumors into zebrafish and studied the clonal evolution in response to drug delivery, also taking advantage of the possibility of using thousands of fish simultaneously, generating a massive amount of data (41).

Stemness is another characteristic of cancer involved in the heterogeneity process and a mechanism that needs to be uncovered. Ignatius et al. selectively labeled differentiated and non-differentiated cells with different fluorophores, being able to sort through FACS the two different populations, revealing divergent expression profiles and behaviors (with the more differentiated cells being highly migratory) after microarray analysis (43). FACS sorting has been also exploited to isolate zebrafish leukemia cells and transplant them into syngeneic recipients allowing the production of monoclonal antibodies and paving the way to new zebrafish cancer models for drug development (44).

It is also possible to prepare libraries of single tumors and transplant them into recipient fish to recapitulate the ITH and to study the effects of drugs (43, 45). The Zebrabow technology based on the multispectral cell labeling for cell tracing and lineage analysis in zebrafish allows the labeling of different tumor clones with different colors and *in vivo* following their migrations and competition in the heterogeneous tissue, also assessing the effects of drug treatments (46, 47).

The optically clear immune-compromised zebrafish “casper” allows the direct visualization of fluorescently labeled transplanted cancer cells and supports the neovascularization and the tumor propagation of heterogeneous clones (48). The “Modeling Approach in Zebrafish for Rapid Tumor Initiation” (MAZERATI) allows to express oncogenes and inactivate candidate tumor suppressor genes using two particular CRISPR vectors, spatially controlling the tumor spreading (49).

#### 2.2.3 Drosophila

Sixty percent of the *Drosophila melanogaster* genome is homologous to humans, with 90% of genes involved in human cancer development having an ortholog in the fly (50, 51). Together with a fast generation time and low maintenance costs, these features contributed to the development of genetic tools to use the fly as a model organism for cancer research (52). The “genetic mosaic technique” lately perfectioned into the “mosaic analysis with a repressible cell marker (MARCM)” creates individually labeled homozygous cells in a heterozygous population, generating cells with a different genotype in a single organism, allowing the researchers to follow the labeled subpopulation destiny (53). MARCM revealed how a single mutated cell in a healthy tissue does not simply overgrow but mostly stimulates the overgrowth and metastasis in the neighbor cells, contributing to cancer progression and probably recurrence (54). The same technique also showed that heterogeneity induces cancer and metastasis by signal propagation (55), molecule exocytosis (56), amino acid release (57), or ROS production (58). Other studies highlighted how different cell populations cooperate in generating tumors: cells mutated in the spindle assembly checkpoints extrude from the epithelium, losing epithelial morphology and adhesion. These mesenchymal-like cells are unable to proliferate but establish a tumor environment by secreting molecules which promote the growth of epithelial cells. So, in this case, epithelial and mesenchymal/mutant cells, which at the beginning are genetically identical, cooperate in the tumor establishment, with the mutant cells unable to proliferate but still activating the others (59–61).

## 3 Human *in vitro* models to study heterogeneity

The biological similarity between animal models and humans has been the basis of the extensive use of these approaches in the study of cancer. However, the failure of many clinical trials and the undeniable evidence of discrepancies in the fidelity of the different models in replicating human physiology generated the necessity of human-derived models.

### 3.1 Organoids

Organoids can be described as microscopic self-organizing, three-dimensional structures, recapitulating many structural and functional aspects of their *in vivo* counterpart organs (62). Biological material such as primary tissues (single cells or tissue chunks), stem cells like adult stem cells (ASCs), induced pluripotent stem cells (iPSCs), and embryonic stem cells (ESCs) can be employed as starting material for organoid production (63). The cells are embedded in an extracellular matrix structure resembling the tissue scaffold and mirroring the physiological milieu to contemplate both matrix influence on cell growth and spatial organization (64); the final result is a heterotypic three-dimensional structure that replicates the multilineage composition of the tissue of origin as well as its molecular, metabolic, and spatial heterogeneity.

It is possible to derive lineage heterogeneity through organogenesis using stem cells (65), while unfortunately, the different cell lineages are not easily preserved in patient-derived organoids (PDOs) obtained from tumor sampling due to the selection of epithelial cells during tissue processing (66, 67). To work around this issue, the introduction of further cell lineages has been applied to organoid models to depict a more complex microenvironment. For example, the desmoplastic reaction represents a neoplastic feature influencing inflammatory response and drug distribution, especially in pancreatic cancer, one of the deadliest cancers worldwide (67); to better investigate tumor–fibroblast interactions in pancreatic ductal adenocarcinoma (68), Biffi et al. (69) developed a co-culture system combining naive pancreatic stellate cells, a precursor of cancer-associated fibroblast (CAF), to organoids generated from pancreatic cancer cells arising from a GEMM spontaneously developing pancreatic cancer. This model was able to reproduce the functional differentiation of pancreatic stellate cells to inflammatory CAF and myofibroblastic CAF elicited by the tumor milieu.

Organoids are also applied to a wide range of tissues and pathologies, e.g., breast cancer (70), liver cancer (71, 72), gastric cancer (73), colorectal cancer (74), prostate cancer (75), and pancreatic cancer (76–78).

In the immune context, Neal et al. (79) were able to establish a patient-derived organoid culture from samples coming from 100 individual patients, covering 19 different tissue sites and 28 pathology subtypes using an air–liquid interface method; however, they encountered major difficulties in preserving the stromal population. The generated organoids mostly recapitulated the parental tumor histology and maintained a complex tissue architecture, but in 70% of the tumors, the stromal myofibroblast population progressively decreased. On the other hand, they observed that PDO-retained tumor-infiltrating lymphocytes (CD3^+^) were integrally embedded in close proximity to the tumor epithelium, macrophages, cytotoxic T cells (CD8^+^), helper T cells (CD4^+^), natural killer (NK) cells, and natural killer T (NKT) cells, which they were able to support using IL-2 supplementation until 60 days of culture. Instead of evaluating resident and tumor-infiltrating immune cells, Dijkstra et al. (80) successfully produced colon-rectal and non-small cell lung cancer PDOs, and later, to study immune response toward cancer and delineate a strategy to develop tailored immunotherapy, they used T-cell populations from peripheral blood to generate a co-culture with organoids; in such a manner, they were able to elicit a specific antitumor immune response mediated by CD8^+^ T cells toward the PDOs. Extensive genetic heterogeneity within cancer cell populations is also documented beyond lineage heterogeneity. ITH, as already presented, provides a substrate for tumor development promoting drug resistance and metastasis. Therefore, it is necessary to model the mutational diversity associated with the branched evolution of clonogenicity, which can be an innate characteristic of PDOs coming from the genetic diversity in the tumor of origin (81) or can be promoted in stem cell-derived organoids through genetic engineering (74, 82, 83). Bolhaqueiro et al. (84) employed colorectal cancer PDOs to investigate the prevalence of chromosomal instability. Single-cell analysis through three-dimensional live-cell imaging and karyotype sequencing highlighted a high frequency of chromosomal instability, which results in aneuploidy and genomic heterogeneity and promotes drug resistance in colorectal cancer.

The highlighted attention to intratumor diversity in molecular studies is promoting an overrunning of personalized medicine and individual clinical plan toward a precision medicine approach that targets heterogeneity itself embracing intracellular modification as well.

In the field of metabolism, three-dimensional organization and multicellular diversification drive the development of differentiated areas and layers resulting in the impaired distribution of nutrients and oxygen with an impact on cell proliferation and metabolism (85); indeed, metabolic heterogeneity affects drug response as well as carcinogenesis (86). Several methodological approaches have been developed for real-time and spatial-resolved metabolism analysis in organoids such as extracellular flux analysis (Seahorse XF analyzer) which allows measuring at the same time and in real time on living cells the oxygen consumption rate (OCR) and the extracellular acidification rate (ECAR) on both cells, spheroids, and organoids in a microplate (87–89). Advanced metabolic flow cytometry analysis such as SCENITH (90) monitors the metabolism through protein synthesis, while MET-FLOW detects rate-limiting enzymes (91). FLIM and PLIM are live-cell microscopy techniques (70, 92–98) that provide unique sensitivity in detecting the metabolic changes occurring during carcinogenesis and anticancer drug response.

While PLIM requires the use of dedicated cell-penetrating phosphorescent O_2_-sensitive probes to perform live-cell microscopy of oxygen, FILM is a non-invasive, label-free, cell-specific direct analysis of metabolism which takes advantage of the intrinsic fluorescence properties of NADH and FAD; an increment in the NADH/FAD ratio observed through metabolic imaging enabled the identification of malignant cells exploiting the Warburg effect (70). These technologies also brought the discovery of metabolic differences between epithelial and fibroblast cells inside an organoid (96) or the detection of intratumor differential response to paclitaxel mediated by the heterogeneous metabolic shift among cancer cell populations (94, 95).

The presence of intratumor multiple stemness niches could be generated as a response to metabolic rewiring due to limited access to nutrients or metabolic changes, which are required to adapt to proliferation rate modifications. Sundar et al. (99) studying cancer stem cell populations (CSCs) in glioblastoma PDO noted that therapeutic resistance is driven by altered biological mechanisms rather than physical limitations of therapeutic access due to the presence of a highly heterogeneous population of CSCs and cycling/senescent cells. Another study shows that tumor organoids displayed spatial heterogeneity with highly proliferating outer region cells surrounding a hypoxic core of mainly non-stem senescent cells, sensitive to radiotherapy, and diffuse, quiescent CSCs which on the contrary were radioresistant (100).

Ultimately, multiple approaches for the unraveling of tumor heterogeneity have been converged in the recent study of Dekkers et al. With the aim to study tumor infiltration and targeting by engineered immune cells (e.g., CAR T cells), the authors developed BEHAV3D, an organoid-based 3D imaging transcriptomic platform (101). This approach integrates multiple techniques to allow functional single-cell behavior analysis of multilineage organoids with spatial resolution (3D imaging) and integrated transcriptomic profiling.

### 3.2 Microfluidic on-chip models, macro models, and advanced co-culture systems

Although the so-far described strategies provide a valuable complement to traditional preclinical models in the study of tumor invasiveness or drug effects concerning specific DNA aberrations, they lack the representation of the stromal compartment which plays a crucial role in cell spatial distribution, growth, invasiveness, and drug sensitivity (102, 103).

Basically, in the models mentioned above, the environmental context, which could both contribute to the development of tumor heterogeneity or be affected by it, is missed.

A step forward in this direction is represented by bioprinted models (104). Indeed, combining organoids with bioprinting technology could be a promising strategy to mimic the genetic, histological, and functional aspects of cancer heterogeneity, proposing it as a useful platform for personalized therapies (102). The introduction into the system of the extracellular matrix (ECM) with the control of its mechanical properties (e.g., matrix stiffness, architecture, density, protein crosslinking, and fiber network configuration) mimics cell growth, cell proliferation, and differentiation reproducing the surrounding physiological environment for cancer heterogeneity development. This approach allows not only to reproduce the background behind the tumor and tumor microenvironment heterogeneity but also to identify the elements involved in this process, controlling and targeting them.

A crucial element that is still missed also by the bioprinted model is the vasculature which could significantly affect and interact with tumor cell differentiation. In trying to overcome this limitation and to resemble the conditions that surround the development of tumor heterogeneity, many efforts have been done in the field of tissue engineering (103–107). Magdeldin et al. developed biomimetic tumoroids which recreate the spatially different exposition to nutrients and oxygen, allowing to test how the induced cancer cell heterogeneity affects the formation of the vascular network and cancer-invasive capability (108). Gilardi et al. developed a variety of *in vitro* assays and readouts to dissect different extravasation steps involved in the metastatic cascade. The authors highlighted a key role of FAK phosphorylation in trans-endothelial migration validating the results in a metastatic *in vivo* model (109). These results drag a parallel between *in vitro* and *in vivo* fostering the employment of *in vitro* models in the development of new effective antimetastatic treatments.

Another attempt aimed to include the stromal compartment within the study of cancer heterogeneity correlates the development of different morphological phenotypes of tumor cells with the heterogeneity of collagen organization confirming the key role played by the tumor microenvironment (110).

A fundamental component of the tumor microenvironment that develops heterogeneous phenotypes is represented by immune cells, particularly T effector cells, T helper, NK cells, B cells, and dendritic cells (24, 111–113). In the context of immune regulation, many cells are involved such as T and B regulatory cells and medullary thymic epithelial cells (mTECs); however, the most studied are the macrophages and neutrophils (112, 114, 115), known to be able to polarize toward both the anti- and protumor phenotypes. Specific protocols have been developed to recreate *in vitro* the polarization of immune cells, paving the way for the possibility of better mimicking the heterogeneity of the tumor microenvironment (116).

A promising future development is represented by on-a-chip models, which allow complex and dynamic culture systems to mimic the heterogeneity of the tumor microenvironment. Indeed, these models potentially include 3D structures, such as the microvasculature, and chemical or physical stimuli (117). Despite their potential, these platforms have not yet been really exploited for cancer heterogeneity studies, but they could hopefully be coupled with patient-derived models to increase the complexity and the reliability of preclinical studies.

Another aspect of tumor heterogeneity is represented by the tumor surrounding environment including the cell–cell and cell–matrix interactions and their role in the tumor fate.

The most suitable model to elucidate this is represented by microfluidic devices which recreate a controlled 3D structure in terms of matrix composition and stiffness, including patient-derived materials (118) and the addition of chemical and physical stimuli, stromal cells such as fibroblast (109) and mesenchymal cells (119), and vascular compartments (120–123). Through microfluidic models, it is possible to reproduce the recruitment of immune cells (124–127) and their heterogeneous behavior able to develop both pro- and antitumor phenotypes (127, 128).

Aside from the improvement brought by the 3D microfluidic models, there are still some important limitations that need to be overcome. Since the complete recreation of all cellular and structural elements of a physiological environment is still unreachable, it is important to carefully focus on the elements principally involved in the pathway of study (119), trying to include at least the most relevant ones. Indeed, the next challenges consist of increasing the complexity of these models, extending the range of analysis techniques applicable, and promoting the validation through clinical studies to obtain evermore reliable platforms.

The works presented in paragraphs 2 and 3 showed the huge ongoing effort in developing preclinical models including the heterogeneity of the tumor itself and of the tumor microenvironment. We showed that there are different approaches to face this peculiar characteristic of the tumor, from the collection of data from patients to the attempt to recreate the heterogeneity through genetic manipulation or the stimuli and the composition of the surrounding microenvironment. All of them have the same goal of identifying and targeting the drug-resistant tumor subphenotypes responsible for therapy failure.

## 4 Emerging technologies to study heterogeneity: single-cell sequencing and spatial genomic analysis

As it will be better described later, heterogeneity depends on cellular interactions and knowing the rules orchestrating the TME and how different cellular subtypes correlate to the clinical relevance would make a huge difference in improving current therapeutic strategies.

In the past, proteomics and transcriptomics in cancer have been studied at the macroscopic level leveraging techniques that are still very important including bulk DNA/RNA sequencing and flow cytometry. For studies regarding rare cellular populations within the TME, such as immune cells, it was difficult to extensively study the impact of the different immune populations.

In the past, bulk RNA-seq has been incredibly helpful in identifying different tumor mechanisms. The gene expression profiles of deconvolution methods estimating the abundance of cells in a mixed population such as the CIBERSORTx algorithm (129) and xCell (130) have been fundamental in understanding the contribution of each cellular population.

**Figure 3:**
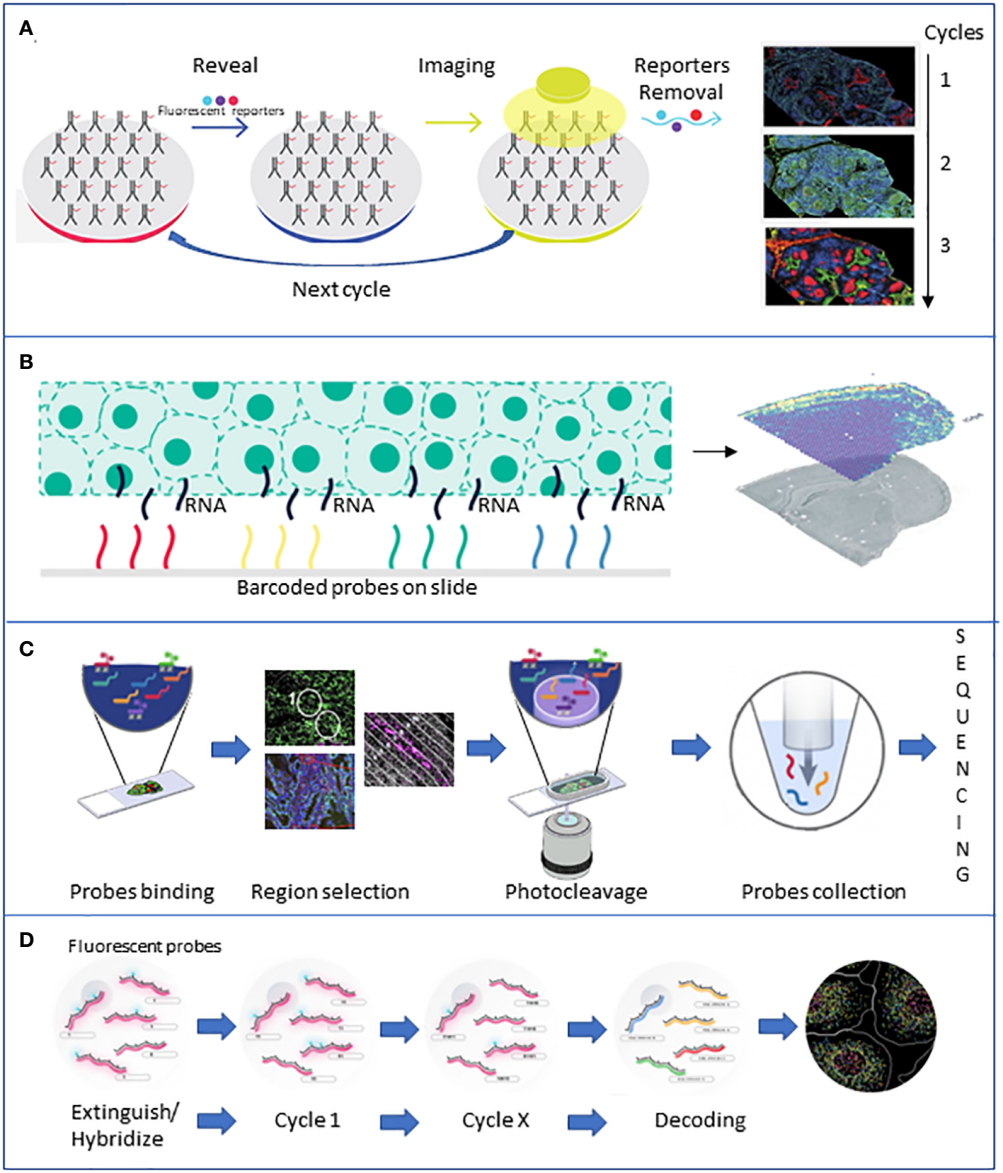
Brief description of the major spatial technologies. **(A)** CODEX is based on a panel of antibodies that binds to specific fluorescent reporters that reveal their position during the imaging phase. At the end of the first cycle of image acquisition, the reporters are detached, and another cycle with new reporters starts. **(B)** 10X Visium is based on slides of barcoded capture probes that bind to the polyA tail of RNAs released from the tissue. RNA is retrotranscribed into cDNA and sequenced. **(C)** GeoMX DSP is based on panels of antibodies or photocleavable barcoded probes. Once an area of interest is selected, a stream of light releases the probes that are lately sequenced. **(D)** MERFISH is based on fluorescently tagged probes that label RNA of interest. Sequential rounds of imaging enable spatial resolution. All the pictures have been adapted from the providers’ web pages.

More recently, with the increased awareness of the role played by TME and the underlying complex cellular interactions, more complex technologies providing single-cell data have been developed. Emerging single-cell technologies and spatial transcriptomics provide new tools to give insights at the single-cell level within tumors and dissect the roles each cell plays in tumor progression (**Figure 3**).

### 4.1 scRNA-seq and spatial analysis

In the last 10 years, the most used technologies to uncover heterogeneity are represented by single-cell RNA (scRNA) and DNA sequencing methods. scRNA sequencing allows the identification of tumor subtypes, definition of cancer cell states, lineage tracing and phenotyping of cellular subpopulation, and differential expression analysis (131).

The new technology applied to cancer heterogeneity allows to detect rare cell subpopulations within the tumor mass, which are very important when it comes to defining the probability of relapse leading to better precision medicine (132). Single-cell data define divergent survival probability improving the clinical prognostic evaluation of each case and therapeutic regimens.

Single-cell profiling of tumor heterogeneity and the microenvironment has been done in many cancer types and metastasis (133) including advanced non-small cell lung cancer, triple-negative breast cancer (TNBC) primary tumor, and paired lymph nodes (134).

Leveraging scRNA, Xue et al. stratified patients into five separate subtypes spatially organized and associated with chemokine networks and genomic features. Remarkably, the authors found that tumor-associated neutrophil (TAN) enriched in the myeloid-cell-enriched subtype was associated with a negative prognosis (135).

Moreover, single-cell transcriptome analysis revealed tumor immune microenvironment heterogenicity and granulocyte enrichment in colorectal cancer liver metastases (131), the role of M2 macrophages in TNBC aggressiveness (136), and the role of TLR4 and TLR8 in TNBC (137). Furthermore, single-cell sequencing coupled with TCR and BCR sequencing allows not only the transcriptomic analysis at the single-cell level but also the possibility to study immune cell clonal expansion. The single-cell level information regarding the immune populations is fundamental to understanding how the diverse immune players will react to different drugs boosting immunotherapy efficacy and the complexity of researchers’ approaches to design novel and more effective combinatorial treatments.

Huipeng Li et al. were able to exploit the single-cell approach to discriminate into subgroups presenting divergent survival probability tumors that were previously assigned to single subtypes through bulk RNA-seq (138). The single-cell technology allowed Wai-Hung Ho et al. to explore the interrelationship between liver cancer stem cell markers reporting new subpopulations of cells and novel stemness-related genes (132).

Single-cell sequencing revolutionized the cancer field providing detailed information at the cellular level. However, given the procedure used to prepare the single cells, the spatial data and all the information regarding the hierarchical structure and how the cells are distributed in the TME are not included in the output.

Digestion of solid tumors characterizes the single-cell RNA sequencing (scRNA-seq) protocol eliminating spatial information and the organization of individual cells in the neighborhood. Tissues are characterized by hierarchical structure organizing how the cells composing the tissue are localized reciprocally. Spatial localization is fundamental in defining cellular interactions and tumor progression. In fact, clones, subclones, immune cells, endothelial cells, and stroma localize in different districts within a tumor tissue, and the spatial information can be used as predictive of therapy response.

Spatial phenotyping allows the combination of various markers within the same tissue slide underlying novel patterns and correlations that would not be evident with non-spatial technologies. A comprehensive overview describing the differences in spatial technologies has been extensively reported (139).

PhenoCycler CODEX, NanoString GeoMx Digital Spatial Profiler (DSP), CosMx, 10X Visium, and MERFISH are among the most used technologies which allow a spatial analysis.

Meyer et al. used the highly multiplexed immunofluorescence imaging technology CODEX to generate a tissue atlas of inflammation in the context of ulcerative colitis compared with healthy tissues. The authors characterized the cell types, cell–cell contacts, and cellular neighborhoods highlighting that cellular neighborhood dictates the functional states of the cells composing the tissue. In addition, this analysis was able to identify different inflammatory cell subsets and spatial neighborhoods peculiar to patients treated with TNF inhibitors, paving the way for targeting specific cellular niches responsible for resistance (51). Spatially resolved data provided insights into ITH allowing phenotype tracking and clonal evolution within tumors. In this context, Rovira-Clave et al. realized the *in situ* tracking of barcodes in small cell lung cancer tumor microenvironment coupling epitopes for imaging (EpicTags) and multiplexed ion beam imaging (EpicMIBI) (140). This approach promoted the ITH spatial investigation interrogating both cell-intrinsic and cell-extrinsic events leading to therapeutic resistance. Hajiran et al. compared survival outcomes to patterns of immune cell distributions defined by spatial analysis in metastatic clear cell renal cell carcinoma (ccRCC). In this study, augmented macrophages together with the decrease in Th1 presence within the tissues correlated with both poor overall survival and worse patient outcome (141). These pieces of evidence support the importance of spatial analysis of immune cells in the tumor microenvironment for the future assessment of clinically relevant associations for improved patient treatments.

Another use of spatial technology is the study of the tumor microenvironment profile to identify potential biomarkers to predict clinical outcomes. Within the new technologies for spatial analysis, 10X Visium and NanoString GeoMX DSP are the most used and often combined (139). The first is based on fixed tissues permeabilized to allow the RNA capture through oligo-dT overhangs, which will be later reverse-transcribed and sequenced, while the second is based on regions-of-interest selection guided by fluorescence methods. Bullman’s group exploited the Visium to study the tumor microenvironment in OSCC and colorectal cancer, showing a spatial heterogeneity in the microbiota with bacterial communities populating the less vascularized and highly immunosuppressed area. Also, they showed that cancer cells infected with bacteria can recruit myeloid cells into the bacterial regions (142).

On the other hand, the GeoMX DSP approach allowed Toki et al. to associate different series of expression patterns to immunotherapy response. Remarkably, this study highlights that PD-L1 expression on macrophages correlates with a positive response to immunotherapy that could potentially be used to predict clinical response to immune therapy in melanoma patients (143). In the context of NSCLC, the treatment with immune checkpoint inhibitors does not provide complete benefit in the clinic, and one of the reasons is that there is still a lack of effective biomarkers to stratify the patient for αPD-1 treatments. The exploitation of the DSP technology showed that the correlation of CD44 levels exclusively in the tumor compartment was associated with a positive response to immunotherapy excluding the immune cells from the analysis. This type of compartmentalized analysis on only the tumor’s immune compartment is peculiar to the DSP workflow and very useful for immunotherapy response biomarker discovery. Leveraging this, Moutafi et al. identified a novel promising biomarker to predict NSCLC sensitivity to αPD-1 therapy (143), while Rimm’s group quantified 39 immune parameters simultaneously in four tissue compartments, correlating overall survival with a high count of CD56^+^ immune cells (143). Remarkably, Hwang et al. recently applied the power of DSP in the context of pancreatic cancer, identifying a new neural-like malignant progenitor enriched after chemo- and radiotherapy, associating it with poor prognosis (144). Nirmal et al. took advantage of the possibility of studying boundaries between different cell populations, identifying a spatially restricted suppressive microenvironment along the tumor stroma boundary in cutaneous melanoma (145).

A recent upgrade of the GeoMX DSP which provides cellular-level data is the CosMx, which brings the spatial analysis to the next level, allowing the localization of RNAs at the subcellular level. Despite being a very novel and recent technology, CosMx has been exploited already by Beechem’s group who analyzed 980 RNAs in non-small cell lung and breast cancer, identifying 10 unique tumor microenvironments inside the cell, proving the presence of spatial heterogeneity inside a single cell (146). A similar output has been obtained by Xia et al. using a different technology called MERFISH, based on a combination of imaging and *in situ* hybridization. The authors determined the subcellular compartmentalization of RNAs and identified populations that are cell cycle-dependent and independent inside the same cell (147).

One major challenge in spatial transcriptomics is the resolution of the data, as the number of cells within a single spatial location (also known as a “spot”) can range from a few to several hundred. This variability can make it difficult to accurately assign cell types and identify spatial patterns of gene expression. Various approaches have been developed to overcome these limitations, such as the use of supervised learning approaches and leveraging cell type profiles learned from scRNA-seq data (148).

The huge amount of data generated by spatial transcriptomic technologies requires new computational methods for the storage and annotation of spatially resolved single-cell data. Another challenge is the integration of gene expression and spatial information. Traditional scRNA-seq techniques do not capture spatial information, so methods have been developed to integrate scRNA-seq data with spatial transcriptomics data (149, 150), as recently reviewed by Longo et al. (151). However, these methods can be complex and may not always produce reliable results (151).

There is also a need for robust downstream analysis tools that can extract biological signals from raw spatial transcriptomics data and identify the spatial organization and cell–cell communications. Some of the existing tools may be limited by technology-specific biases or may not be suitable for all types of spatial transcriptomics data. Computational methods are emerging (152). Recently, new efforts in the field promoted the generation of new methods of analysis of spatially resolved single-cell data allowing a more accurate cell-type annotation and phenotyping such as Seurat, stellaR, SpatialDecon, and tangram, summarized elsewhere (153). These types of tools favor the discovery of new types of cells in spatially resolved datasets at a single-cell level, a fundamental step in the definition of tissue hierarchy and underlying biological processes. The reader is referred to Dries et al. for a review of the art of spatial transcriptomic data downstream analysis methods and pipelines (154).

This recent progress is just an anticipation of how single-cell RNA sequencing and spatial transcriptomics techniques will play an essential role in the next future in incorporating tissue architecture with transcriptomics data. The ability to see what is going on at the reface of a tumor-infiltrated tissue and its healthy neighbors at the RNA and protein levels or the possibility of visualizing what a particular group of immune cells express when they are interacting will greatly impact our prognostic abilities and our knowledge on heterogeneity.

Being able to combine the data coming from different techniques will require interdisciplinary teams composed of molecular biologists, pathologists, and wet lab and data scientists. Yet, the information implemented in the clinical system will unlock enormous achievements in the field of targeted therapies to overcome resistance to treatment and prevent metastasis.

### 4.2 Bioinformatics and computational modeling

**Figure 4:**
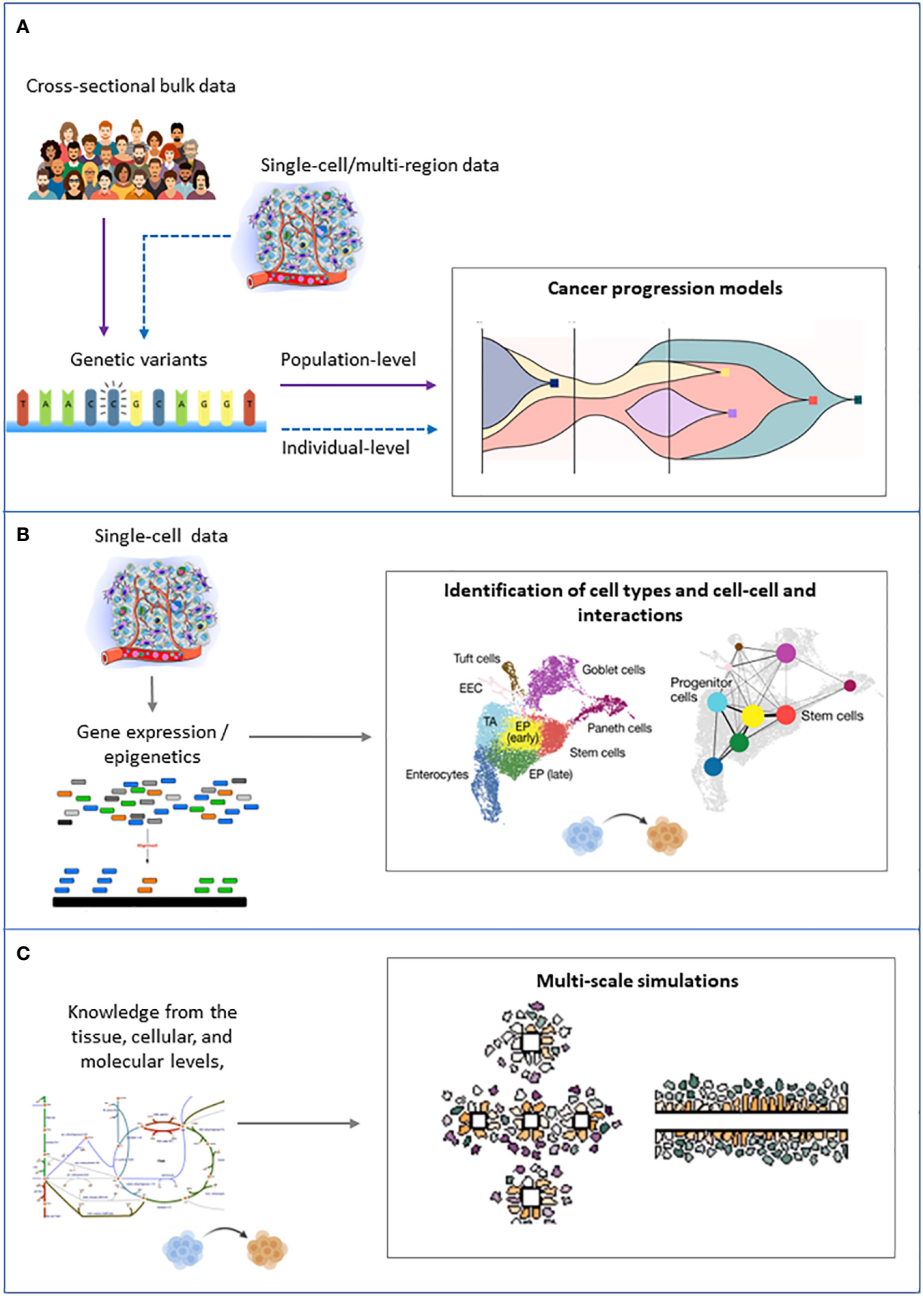
Schematic representation of the inputs (left) and outputs (right) of the main families of computational approaches to tackle intratumor heterogeneity. **(A)** Cancer progression models. **(B)** Clustering of single cells. **(C)** Multiscale modeling and simulation.

The computational approaches that are fundamental to tackle ITH can be grouped into three families: 1) approaches that try to infer the tree of clonal evolution from sequencing data, 2) approaches that aim to identify the different cell types in a cancer cell mixture from single-cell and/or spatial transcriptomics and epigenomics data, and 3) knowledge-based models that aim at simulating the dynamics of cancer cell populations (**Figure 4**).

#### 4.2.1 Inference of cancer progression models from sequencing data

Genomic alterations [i.e., single-nucleotide variants (SNVs); structural variants, such as insertions and deletions; and copy number alterations (CNVs)], which can be identified by opportunely processing next-generation sequencing (NGS) data (e.g., DNA-seq, RNA-seq, or ATAC-seq) of tumor samples, can be used to track tumor progression. The philosophy and clinical implications of this approach are reviewed elsewhere (3).

The basic idea is that the positively selected (i.e., functionally advantageous) genomic alterations (i.e., drivers) identified in every cancer cell represent the clonal trunk, whereas those identified in a subset of cancer cells defined the coexisting (sub)clones.

In the last years, a plethora of bioinformatics tools have been developed to exploit mutational profiles of cancer samples to reconstruct a model of cancer evolution, either at the population or at the individual level. Genomic alterations can be used to characterize and track down tumor progression through NGS (3).

Population-level models are typically inferred from cross-sectional bulk sequencing data, in which one sample is available for each patient. The objective is to infer a unique progression model for the patient cohort under study, which usually represents a specific tumor (sub)type (155). In the final model (which can either be trees or direct acyclic graphs), edges represent the most likely trends of accumulation of genomic alteration for that specific tumor (sub)type and can be used to both predict the next evolutionary steps and to stratify patients in risk groups.

Individual-level models aim to reconstruct a personalized progression model for each individual. These models ideally require multiple measurements for the same tumor, which can either be:

- single-cell sequencing data, collected either at single (156) or multiple time points (longitudinal), e.g., from patient-derived cell cultures, xenografts, or organoids.
- multiregion bulk sequencing data (156).

In this case, the output model (a mutational/clonal tree) depicts the evolutionary history of a single tumor and, in the case of longitudinal experiments, allows one to assess the impact of external interventions, such as therapies.

The methodology at the core of these tools is generally based on traditional sequence-based phylogenetics (157, 158) or on Bayesian/maximum-likelihood statistical frameworks (159–161). The main differences between the two approaches are illustrated here (162).

Fundamental data preprocessing steps are needed upstream of all aforementioned methodologies and differ according to the specific experiment type (single cell or bulk) and data type (DNA, RNA, ATAC, and related technology/protocol). A non-exhaustive list includes the correction of sequencing reads; the correction for purity, ploidy, absolute copy number, and mutation multiplicity; variant calling; and estimation of variant allele frequency (VAF), which must be converted into a cancer cell fraction (CCF) taking into account gene copy numbers. To maximize the sensitivity and specificity of calling clonal and subclonal mutations, the PCAWG Evolution and Heterogeneity Working Group and the PCAWG Consortium used an ensemble approach integrating the output of alternative algorithms (163). However, the number of tumor regions sequenced and the depth and purity of what is sequenced largely affect the ability to distinguish truly clonal from pseudoclonal mutation. Strong tumor sampling bias, high levels of technical noise, and biological variability also hinder the robust inference of cancer progression models. To mitigate this problem, a recent work has proposed to use a transfer learning approach to infer from multiregion data multiple patient evolutionary models simultaneously, seeking to maximize their structural correlation (164).

It remains an open question whether cancer progression models can truly predict the likely course of tumor progression or whether the occurrence of neutral evolution and drift may limit the ability to predict a tumor’s next step. To address this question, Diaz-Uriarte and Vasallo (165) analyzed four different approaches and concluded that these methods can predict only with moderate success and only under representable fitness landscapes and with very large sample sizes, but even perfect algorithms might not work if intrinsic evolutionary unpredictability is large.

#### 4.2.2 Clustering and lineage inference from single-cell transcriptomics and epigenomics data

Phenotypic data at the single-cell level allow variability due to the environment and interactions among cancer subclones as well as with the other player in the TME to be considered. In this regard, unsupervised machine learning methods (clustering) on single-cell RNA-sequencing data were successful in unraveling the composition in terms of cell phenotypes of a cancer mixture (166, 167). RNA velocity and cell lineage reconstruction might also be employed to investigate the similarity and dynamics of cancer cell types (168). Computational methods to infer cell–cell communication events from scRNA-seq data have also been proposed (169), even focused on the identification of metabolic cooperation phenomena (170).

The noisy nature of single-cell RNA-sequencing data requires *ad hoc* preprocessing steps. To this aim, the best practices (171) and tools implementing them such as Seurat (148) and Scanpy (172) are now well established. A preprocessing stage that requires special care and that is largely debated is denoising of scRNA-seq data, as reviewed by Patruno et al. (173) This step becomes fundamental when the aim is to identify metabolic subpopulations from scRNA-seq, as demonstrated by Galuzzi et al. (174).

Approaches to integrate scRNA-seq data with other -omics have recently emerged. For example, CONGAS integrates bulk DNA and single-cell RNA measurements from independent assays to jointly identify clusters of single cells with subclonal CNAs and differences in RNA expression. The opportunity provided by the latest technologies to simultaneously profile intranuclear proteins, chromatin accessibility, and gene expression in single cells is pushing forward the need for single-cell multiomics data integration (175, 176). In particular, methodologies for handling sequencing data that simultaneously measure gene expression and chromatin accessibility in the same cell are increasingly being proposed (177–179); for instance, statistical and machine learning methods for spatially resolved transcriptomics data analysis are currently being developed and have been previously reviewed (179).

#### 4.2.3 Multiscale modeling and simulation

The data science approaches described above cannot identify mechanisms nor probe whether the correlation is causal. On the contrary, multiscale modeling in systems biology allows the behavior at the larger scale to emerge naturally from the collective action at smaller scales.

Multiscale models integrate a priori knowledge from the tissue, cellular, and molecular levels and can simulate complex cell–cell interactions and emerging population-level dynamics. These models are generally based on ordinary differential equations that can simulate the integral response of the tumor to pharmaceutical interventions (180) but fail in capturing spatial phenomena. For the study of invasion and metastasis, models based on partial differential equations or agent-based models are applied (181). In particular, agent-based modeling is the most suitable framework to model ITH because it can describe the dynamics of a large number of heterogeneous and interacting agents (i.e., cells or clones) that act autonomously in an environment according to certain rules. Agent-based models have been used to study the differentiation of cancer stem cells (182), clonal evolution (183, 184), and interaction between different cell types (185) and different metabolic phenotypes (186).

While multiscale models can provide unprecedented insight into mechanistic detail, they are computationally expensive and require a large number of unknown parameters to be defined. Real-world data are generally used only to fit the model parameters, for example *via* approximate Bayesian computation (ABC), whereas attempts to include omics data directly as parameters of the multiscale model are still in their infancy (187, 188).

The definition of new computational frameworks that bridge the gap between top-down approaches (close to the data but far from the mechanisms) and bottom-up approaches (close to the mechanisms but far from the data) is a key objective to enable the reconstruction of digital twins that integrate biological knowledge and population data with personalized data. In this regard, machine learning and multiscale modeling can naturally complement each other to create robust predictive models that include physicochemical constraints (189). Data generated by mechanism-based simulations can also supplement training data for machine learning models.

## 5 Tumor heterogeneity, drug resistance, and clinical outcomes

It is well established that tumors with high levels of ITH may predispose patients to worse clinical outcomes (189). The main reason is that ITH implies the coexistence of subclones with different genetic, epigenetic, and metabolic endowments.

On the one hand, this can expose to a greater probability of achieving in at least one tumor cell population, with subsequent genetic and epigenetic alterations, a molecular combination that leads to the acquisition of a physiological alteration determining a clonal expansion *in situ* or dissemination, thus contributing to tumor progression.

On the other hand, it guarantees the tumor greater adaptability to environmental changes, possibly induced by exogenous factors such as pharmacological treatment, and therefore, increases the fitness and survival of cancer cells and intrinsic resistance to therapeutic treatments that determine tumor relapse.

Resistance is considered intrinsic when conditions for escape from the drug response are already inherent in the treated tumor. It is considered acquired when the treatment itself activates adaptive mechanisms that lead to resistance.

An example of intrinsic and acquired resistance is found in tumors associated with hyperactivation of the epidermal growth factor receptor, EGFR, which transduces the mitogenic signal in response to the growth factor by activating Ras proteins and their cellular effectors. The oncogenic activation of this receptor may be due to gene amplification (copy number variation) increasing its expression level, deletions (truncations of regulatory regions as in EGFvII and EGFR carboxyl-terminal deletions), or point mutations (substitutions of residues critical for the function) that make the receptor constitutionally activated (190, 191).

Although many effective treatments are available (192, 193), there is also a very high percentage of patients who after a few months of treatment manifest resistance and tumor relapse (194) due to on-target mechanisms dependent on the coexistence of cell populations with different sensitivities to the drug (present before the treatment or acquired by selective pressure) (195) and/or off-target mechanisms depending on the oncogenic activation of other genes and proteins (196, 197). Other examples of drug resistance due to heterogeneity can be found in the study of Blaquier et al. (198) and in other studies (192, 194, 199).

Another example of ITH leading to drug resistance concerns the presence of cancer stem cell niches in the tumors, with a small population of cells endowed with stemness properties, including enhanced capacity for self-renewal cloning, the undifferentiated state combined with differentiating potential, long cell cycling, genome repair abilities, peculiar energy metabolism, ability to educate the neighboring cells to provide nutrients although highly resistant to lack of nutrients and hypoxia, and ability to collaborate in the elusion from the immune system (200). Cancer stem cells confer high plasticity to the tumor and contribute to drug resistance with multiple mechanisms, for instance by remaining quiescent during chemotherapeutic treatment specifically targeting proliferating cells and then regrowing for repopulation or efficiently repairing DNA damage induced by some chemotherapeutic agents, such as platinum drugs and alkylating agents.

In summary, ITH and the response to drug treatment are interdependent phenomena. On the one hand, pretreatment tumor heterogeneity is mainly responsible for intrinsic drug resistance and relies on multiple mechanisms including the presence of cells in the tumor site expressing elements that bypass target inhibition because they promote aberrant downstream signaling (i.e., Ras oncoproteins in EGFR-hyperactivated tumors), or cells expressing MDR pumps, or cells endowed with potentiated DNA repair system, or cancer stem cell niches. On the other hand, the adaptation to pharmacological treatment, particularly if directed against a specific target as in the context of precision medicine, feeds the ITH and predisposes it to drug resistance negatively affecting clinical outcomes.

Historically, heterogeneity has been mostly associated with mutations, and only recently, heterogeneity has acquired an enlarged origin including selective stimuli from targeted therapy and the tumor immune microenvironment. All these factors have been known to be responsible for the heterogeneity mechanisms, yet the emergence of new features peculiar to cancer cells only has been associated with the generation of neoantigens correlated with a positive outcome, especially in immunotherapy. Thus, understanding how to modulate the immune response by controlling heterogeneity should be further investigated to better develop targeted therapies.

## 6 The role of the tumor microenvironment in heterogeneity

Selective death in the TME can lead to heterogeneity. To explain this concept, we will leverage a conservation biology theory by Martin and Sapsis. The “pyrodiversity promotes biodiversity” theory first proposed that fire promotes biodiversity by generating heterogeneous ecological niches and inducing species to adapt to environmental changes (201, 202).

We can look at the cancer ecosystem in the same way. Usually, selective pressure such as therapy (fire) eliminates all the cells. However, in rare special niches, the conditions for clonal evolution instead of death are presently leading to adaptation through mutation, evolution, and in the end, cancer progression (203).

**Figure 5:**
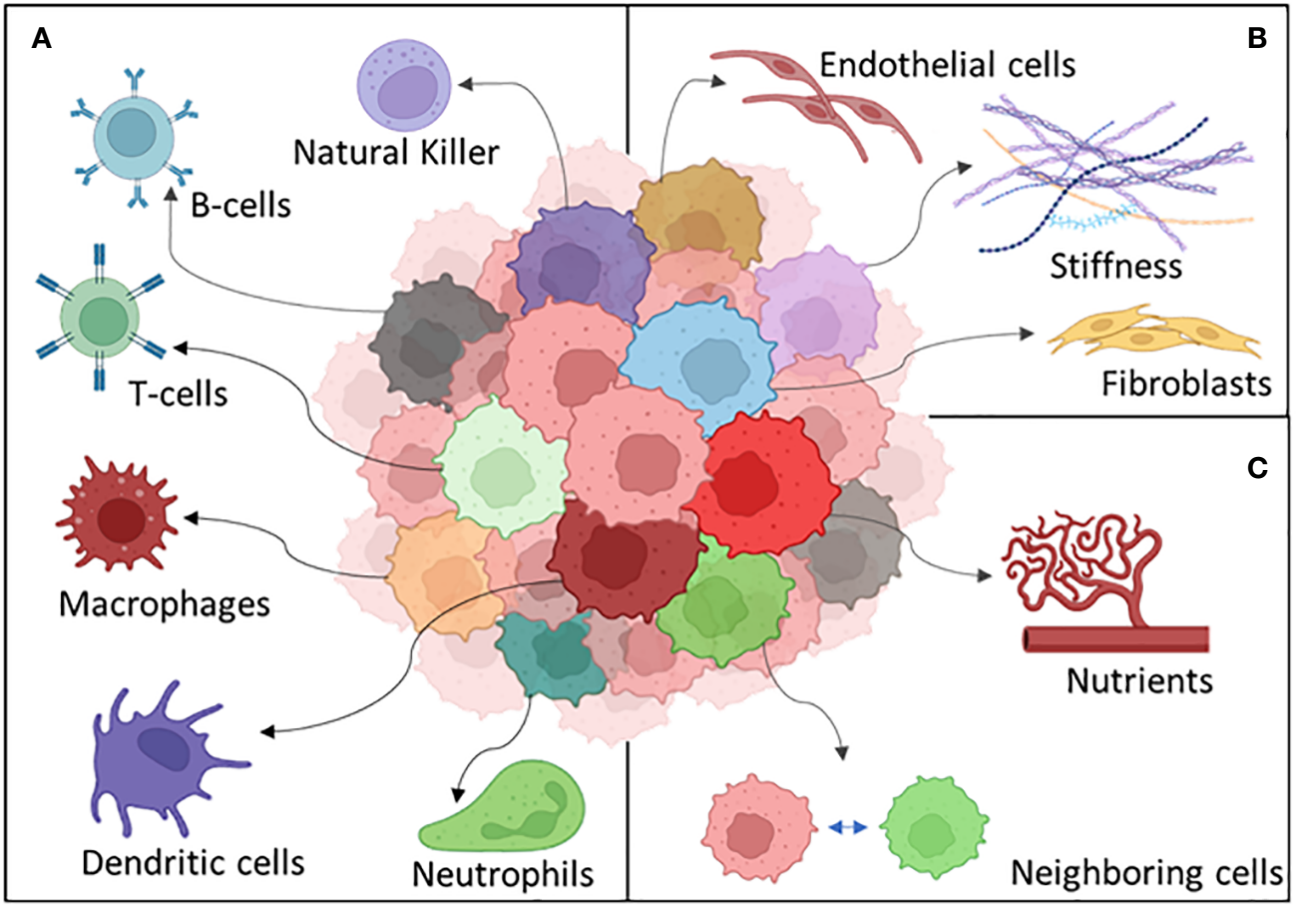
The different components of the tumor microenvironment: **(A)** the immune system, **(B)** stroma, and **(C)** external factors.

Many other different things can happen to a tumor cell exposed to the challenges peculiar to the microenvironment. Stromal cell interaction and immune recognition can be some of the key heterogeneity driving factors which a cancer cell should adapt to survive (204) (**Figure 5**).

### 6.1 Immune recognition and cancer heterogeneity

In the past, heterogeneity has been seen as a negative factor present in tumors correlated to an increased mutational burden, cancer progression, and acquired resistance. In the context of heterogeneity-involved diseases, it is dutiful to mention metastases. Metastases are responsible for more than 90% of cancer-related mortality, and one of the triggering processes is the selective pressure in the TME (205–207). Thus, a deep understanding of heterogeneity underlying these mechanisms will provide the required insight for primary tumor and metastasis eradication.

The advent of improved experimental technologies, such as RNA sequencing, single cells, and spatial analysis of tissues, together with better bioinformatic tools boosted correlative studies between immune profiling, mutational burden, and patient outcomes which will be discussed later in this review.

Neoantigens derived from cancer mutations are one of the keys to unleash an effective and lasting immune response, yet they are derived from mutations that are known to lead to heterogeneity and resistance. A consistent portion of research has been done in the neoantigens field in the last 20 years (3, 163, 208, 209). Yet, the argument is still controversial.

Clinical studies reported a tight association between high tumor mutational burden (TMB) and improved outcomes during immune checkpoint inhibitor regimens. TMB is also reported as a biomarker to predict immunotherapy and chemotherapy efficacy (210).

Ke-Yue Ma et al. (211) characterized in lung adenocarcinoma the ITH of immune response-related genes. They showed that the decrease in the number of neoantigens was correlated with an acquired resistance phenotype. Moreover, the authors reported that MHC-II genes were the common genes shared by the top favorable prognostic pathways supporting that neoantigen presentation by MHC-II may be a positive factor triggering cancer eradication by immune cells.

The improved response to therapy observed in high TMB tumors is also probably due to a broader repertoire of tumor-specific mutant epitopes presented by antigen-presenting cells (APCs) (212) and to enhanced epitope-spreading mechanisms diversifying the ability of the immune cells to recognize multiple targets (113).

Epitope spreading is a mechanism enhancing and diversifying endogenous lymphocyte recognition to new antigens beyond the original one which was the initial target antigen. This mechanism can involve intramolecular antigens (recognition of epitopes within the same protein) or intermolecular ones (involving other proteins) and can lead to enhanced cytotoxic T-cell activity and anticancer antibody production by B cells (213). Although epitope spreading positively correlates with patients’ responses representing an important predictive marker (214), it correlates also with side effects due to T-cell recognition of autoantigens and to the expansion of the autoantibody repertoire (215, 216).

Epitope spreading is an incredibly powerful mechanism triggering parallel immune recognition and also autoimmunity. Its fine regulation makes the process very complex; thus, more efforts need to be made to be able to leverage it therapeutically to overcome autoimmunity.

Reuben et al. (217) studying lung adenocarcinoma showed that although an increasing variety of neoantigens promote a wider heterogeneity in the T-cell receptor repertoire, it also correlates with impaired survival and tumor relapse. There can be many reasons why more neoantigens lead to negative outcomes. One of the hypotheses can be designed by dragging a parallel between cancer cells and pathogens. The mechanism underlying cancer immune escape leveraging heterogeneity can be associated with the ability of some pathogens, such as *Borrelia burgdoferii*, to escape immune recognition by overwhelming the immune system with antigens that are not determinants of pathogen eradication (218).

Other immune cells beyond lymphocytes are involved in reshaping the heterogeneity of the TME. Clinical cancer stage and metastatic tumor burden are linked to ineffective immune response and increased immune suppression due to myeloid cell infiltration in different tumor types including pancreatic cancer (219) and breast cancer (220). In line with these findings, Zhang et al. (131) revealed tumor immune microenvironment heterogenicity and granulocyte enrichment in colorectal cancer liver metastases.

A common model for cancer heterogeneity is the “cancer stem cell” in which tumor-propagating cells have the same genetic mutations as the differentiated cells but are blocked in a different maturation stage. This is known to correlate with poor patient outcomes, resistance, metastasis, and immune suppression (221, 222).

Cancer cells can leverage epigenetic reprogramming to increase stemness and escape the immune system (223). Consistent with this, Miranda et al. (224) described a negative parallel correlating decreased survival, impaired T cells, natural killer cells, B-cell immune infiltration, and increased stemness. Barker et al. (225) identified cancer stem cells in the small intestine and colon using Lgr5 as a marker opening the door to targeted therapy directly tackling stem cells. This type of antigen could be exploited for targeted immunotherapy. Another way to see stemness is that cancer cells do not upregulate stemness on purpose. Instead, it could be that the cells with stemness characteristics are those that can escape the immune system recognition being the resistant ones. Supporting this, it has been discovered in animal models that NK recognition of metastatic cells in the liver is responsible for dormancy (226) (a quiescent cell state often associated with stemness), highlighting how cancer cells’ interaction within the TME regulates cancer cell survival to modulate their behaviors to escape (227). In another study, it has been highlighted how the macrophage-secreted factor supports pancreatic cancer metastasis by inducing fibrosis (228). Thus, impaired immune recognition by the immune system due to other circumstances can lead to metastatic overt colonization.

The so-called epithelial-to-mesenchymal transition (EMT) is involved in cancer evolution concurring with the development of more aggressive subclones (229). Different studies leveraging RNA-sequencing data correlated EMT-related gene expression profiles with increased aggressiveness. Although these data are very important to reveal the mechanisms involved in the TME, it is unknown if EMT signatures were from cancer or stromal cells given the bulk nature of the analysis. McCorry et al. (230) highlighted EMT signatures in the stromal fibroblast within the tumor microenvironment instead of a change in the cancer cells. Fibroblasts within the TME are not only involved in EMT; Chen et al. (231) showed that tumor-associated macrophages promote EMT and cancer stem cell properties in TNBC. Altogether, these lines of evidence show how important stromal and immune cell heterogeneity is in tumor progression.

In clinical and preclinical work, huge efforts are currently active toward combinatorial therapies leveraging both chemotherapy and immune checkpoint inhibitors targeting the TME (232–235). In this setting, while chemotherapy mediates tumor destruction which leads to the release of tumor neoantigens, immunotherapy keeps the immune system active, targeting exhaustion and suppression. These combinatorial strategies tackle cancer from both angles leveraging neoantigens (generated by mutations) as weapons to train the immune soldiers to fight more efficiently. In solid tumors, high quality and quantity of neoantigens correlate with improved survival and response to immunotherapies (236, 237). In a deeper analysis, a decrease in expressed clonal neoantigens correlates with increased immune infiltration (238) indicating the impact of the immune activity regulating tumor heterogeneity. Altogether, these data show that modulating heterogeneity will be an effective way to have controlled and targeted immunotherapy increasing efficacy while overcoming unwanted side effects.

### 6.2 Stroma heterogeneity in the TME

Other cells within the tumor immune microenvironment can be responsible for selective pressure on cancer cells since they are able to modulate environmental conditions such as cytokines, nutrients, metabolites, matrix stiffness, pH, and redox status.

One of the main populations involved in shaping the TME is the fibroblast. This population of cells is heterogeneous and responsive to different stimuli. CAFs turned out to be responsible for the immunosuppressive microenvironment linked to therapy resistance (239, 240) and metastatic progression (239, 241, 242). Furthermore, fibroblasts are responsible for fibrosis leading to hypoxia, reduced immune infiltration and vascularization, and nutrient deprivation in pre-HCC conditions, leading to HCC cancer progression (243–245).

The heterogeneity of CAFs has been associated with organ-specific metastasis in pancreatic ductal adenocarcinoma (241). Furthermore, in another study, the promotion of cholangiocarcinoma growth by diverse cancer-associated fibroblast subpopulations has been reported (246).

Cancer cells are also able to reprogram CAF gene expression and metabolism (247). More recently, it has been reported that CD10^+^GPR77^+^ CAFs promote cancer formation and chemoresistance by supporting cancer cell stemness (242). In the context of metabolism, cancer-derived exosomal HSPC111 promotes colorectal cancer liver metastasis by reprogramming lipid metabolism in CAFs (248).

Mentioning the interaction between CAFs and immune cells, Krishnamurty et al. (249) reported that LRRC15^+^ myofibroblasts dictate the stromal setpoint to suppress tumor immunity.

Endothelial cells are fundamental players in the TME being involved in angiogenesis and adhesion molecule expression on the vasculature regulating immune cells and nutrient trafficking. The endothelium is a heterogeneous tissue, and different expression profiles have been reported characterizing endothelial cells from different organs. Ultimately, in order to generate more effective targeted approaches against cancer progression and metastasis, we need to take into account the different endothelial barrier properties contributing to organotropism metastatic behaviors of different tumors (250).

The cellular component is not only involved in the control of tumor heterogeneity. The sensing compartment composed of the matrix is important as well in regulating how the cells sense the surrounding environment. The tumor matrix stiffness plays a role in the mechanotransduction of tumor cells involving integrins signaling to modulate how cancer cells can feel the microenvironment.

It has been demonstrated that different stiffness leads to modified gene expression (251–255). Stiffness reduction has been proven to improve bevacizumab response in metastatic colorectal cancer (256). Changes in other parameters such as pH have also been reported to improve immunotherapy efficacy (257).

This plethora of parameters present in the TME modulates in turn the immune system leading to inflammation (14) or immunosuppression (114, 115, 258). These two types of TME determine the fate of cancer cells (229) selectively pressuring them to evolve or perish. Learning how to control the mechanisms underlying heterogeneity will provide knowledge to leverage this information therapeutically. The final aim will be modulating heterogeneity to make cancer cells seen by the immune system to unlock immune recognition.

## 7 Discussion and conclusions

The recent awareness of the importance of heterogeneity in the development and establishment of tumors opens up new possibilities for understanding tumor development and—in perspective—improving and personalizing therapeutic approaches to tumors. Different technologies, some of which are discussed in this review, open hitherto unexplored windows to the understanding of tumor biology at the single-cell and spatial levels.

In particular, single-cell techniques enabled the discovery of cellular differences that usually get lost during bulk RNA-sequencing sampling methods, helping the scientific community to understand how different cellular populations express different sets of genes. The spatially resolved transcriptional analysis revolutionized the study of heterogeneity allowing transcriptome analysis without losing the spatial organization of tissue architecture.

Both single-cell and spatial transcriptomics can generate heavy databases of data, challenging the scientific community in developing new ways to analyze, store, and integrate the data. Multiomics technologies with the promise to deliver high-throughput genomic and epigenetic molecular data in parallel will combine RNA, DNA, and ATAC sequencing technologies for more comprehensive studies. For instance, scATAC-seq allows epigenetic studies, lineage tracing, and genomic regulation, providing insights on chromatin accessibility, and the full-length mRNA profiling in single cells exploited to study the alternative splicing (259). The processing of millions of cells is required to detect a rare subpopulation of cells, and this can be achieved by single-cell sequencing (combinatorial indexing). Accordingly, integrating data of a widely diverse nature in terms of dimensionality of data (a few proteins, hundreds of biochemical or imaging features, the whole transcriptome) and experimental approach (targeted, hypothesis-driven *vs*. exploratory genome-wide) is probably the primary challenge. Therefore, it is necessary to use computer and computational techniques for an in-depth analysis of individual data (i.e., transcriptomic, proteomic, or any -omics data) and also to integrate and structure data related to different layers of biological complexity: the final aim is to describe the emergent properties derived from the interaction of the system components and those that cannot be derived by the mere knowledge of the properties of the individual components (260). For instance, because of the special role played by metabolism in orchestrating cellular activities (261), the simulation of computational models of metabolism acts as an integrator able to explain at the system level the phenotypic properties of cellular systems and even their interaction (262). The modeling approach is somehow complementary to the artificial intelligence/machine learning approach, which excels in differentiating and stratifying patient populations but so far has proven less suitable for identifying the laws governing complex biological phenomena (261, 263). Wiring together the analytical and correlative ability of machine learning with the ability of mathematical models of metabolism and other cellular functions to structure biological information could allow a quantum leap in our understanding of tumor heterogeneity.

The generation of enhanced computational models to prioritize and predict therapeutic efficacy leveraging cancer molecular profiles has been recently developed.

An example of successful integration of multiomics data together with phenotypic and therapeutic response profiles falls into the computational strategy called pharmaco-pheno-multiomic (PPMO).

This model allowed the establishment of novel complex biomarker profiles to predict prospective therapeutic regimen response in acute myeloid leukemia and ovarian tumor cohorts (264). These strategies already demonstrated their extraordinary potential in predicting therapeutic response in human tumors and need to be further exploited on different cancer types and broader cohorts in the future.

**Figure 6:**
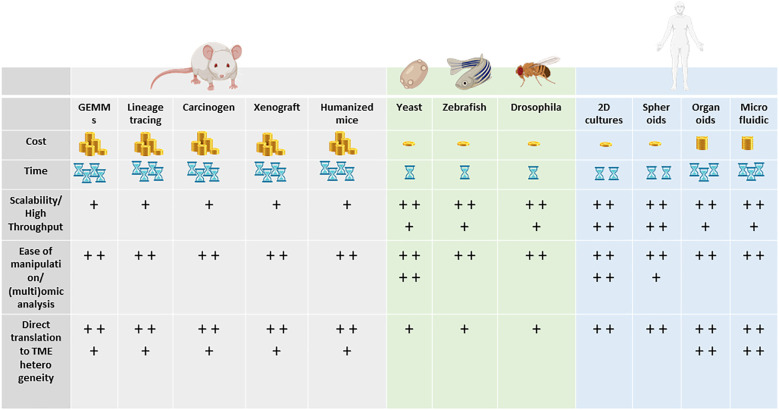
Key properties (cost, time, scalability, ease of manipulation, direct translation to TME heterogeneity) of the different models highlighted in this review. From left to right: murine models, non-murine models, and human models. + = low, ++ = medium, +++ = good / high, ++++ = excellent / very high.

The availability of different non-human models of tumor heterogeneity is of great value since it will allow us to experimentally test in an iterative cycle the computer-generated predictions (265). **Figure 6** graphically summarizes some of the key properties of different models highlighted in this review. Despite lacking some aspects of the complexity present in mammalian systems, non-murine models can be exploited for preliminary screening to answer precise biological questions. Zebrafish, *Drosophila*, and yeast, among others, have already proven their efficacy in recapitulating basic and conserved biological mechanisms, coupling this with the possibility of collecting thousands of data points in a quick and cheap way. Although the way of life of yeast is unicellular, yeast cells demonstrate the ability to coordinate to form multicellular communities with specialized subpopulations, as what happens in a tumor mass where, from a single progenitor, many cells arise and specialize to survive. An extra layer of complexity concerns the signaling between different colonies, which induces metabolic reprogramming (38) to maintain the identity of the single colony. The study of this intra-and intercolony crosstalk could uncover evolutionarily conserved mechanisms that can be targeted to prevent the establishment of a colony/tumor mass in a new environment. With 90% of genes involved in human cancer development, fast generation time, and low maintenance costs, *Drosophila* is perfectly suited to study the basic mechanisms of cancer heterogeneity. Zebrafish is a unique model that allows extensive characterization of the mechanism of clonal evolution, also allowing the identification of dominant drivers. Transgenesis, transplantation, single-cell functional assays, and live imaging can provide an economical and large-scale *in vivo* screening tool which provides statistically relevant data to complement focused studies done in mice or humans, as published by Smith et al. (44), where a zebrafish study revealed that one in 100 leukemia transplanted cells was able to drive tumor growth, a higher number than expected if compared to mouse studies.

Models such as yeast and 2D cultures excel in their ability to perform genetic and environmental modulation of growth conditions and in their ability to run omics analyses, including single-cell transcriptomics. Although the clonal nature of cell lines grown as monolayer offers high reproducibility and low intrinsic heterogeneity, their high intrinsic plasticity allows fostering of different phenotypes within the same cell line, for instance by using inflammatory cytokines and growth factors (266).

Murine models, on the other hand, provide a complex systemic platform to evaluate biological mechanisms and both drug efficacy and toxicity.

Since their development, mouse models have become more sophisticated and accurate in replicating human tumors including *ad hoc* models to study heterogeneity.

Among these, GEMMs and syngeneic models are exploited for their high reproducibility and flexibility, yet mouse tumors have different evolution routes if compared to human ones. This results in decreased genetic heterogeneity which penalizes the clinical translational relevance of these models. To overcome the species-specific issues, human cell line-derived xenografts (CDXs) are largely leveraged due to their fast and standardized growth.

Although CDXs are composed of clonal populations, their level of genetic heterogeneity does not compare with human tumors. Immortalization and *in vitro* clonal selection can be the cause of genetic drift. In addition, the lack of cell–cell interactions within a 3D human microenvironment limits the clinical predictivity of the findings obtained by exploiting human cell lines.

The type of mouse model that mostly translates the results of the experiments to clinical response is the PDX which maintains the heterogeneity of the patient’s primary tumor especially when used at low passages (less than 10). PDXs have been successfully used in the context of personalized cancer treatment as an investigational platform for therapeutic decision-making (267).

Given the predictive capacity of PDXs in clinical translational studies, the urgent need to leverage them to predict immunotherapy outcomes emerged quickly. In fact, to study immune therapeutic regimens with PDXs, the animal host requires a functional immune system of human origin. The implementation of humanized mice together with low-passage PDXs in the same study allows for both the clonal heterogeneity of the human tumor and the immune microenvironment to be reconstituted. One of the limitations is the graft-*versus*-host disease (GVHD) happening when the engrafted human immune cells are from a different donor with respect to the PDX one. To avoid unwanted GVHD which will impair the validity of the study results, human leukocyte antigen (HLA) matching strategies between the donors should be done. Ideally, autologous models can be proposed. However, this option is still very limited given the lack of primary matched tumor and immune material from the same patient.

In basic science and preclinical settings in the attempt to replicate tumor heterogeneity, animal models have been extremely powerful and extensively used. Searching for faster, cheaper, and more ethical models to evaluate in high-throughput entire libraries of drugs and the divergence of these models in mimicking some aspects of human biology paved the way for the establishment of human-derived advanced *in vitro* models.

Organoids and on-chip microfluidic models using human-derived cells are great tools for retaining tumor heterogeneity, especially when employing low-passage primary human cells. These *in vitro* models are suitable for high-throughput screening of multiple therapeutic combinations or as a platform to investigate human key molecular pathways allowing the analysis of complex cell–cell and cell–matrix interactions in biochemically and biophysically controlled conditions. In addition, the reduced time in providing the results makes the *in vitro* humanized models ideal for preclinical studies adding value to the animal models, which, on the other hand, are still essential for systemic and toxicological studies. Despite the tremendous advances in modeling, when the preclinical use of these models is required, researchers need to consider that different models display variable fidelity to human tumor biology. Organoids represent the best option to preserve tissue heterogeneity using *in vitro* culture. By carefully choosing the protocol of production, it is possible to retain molecular, spatial, and metabolic heterogeneity of the tissue of origin. However, there are limitations regarding the cell lineage that can survive inside the organoids during culture (e.g., immune cells), and the sampling of the tissue of origin can affect the heterogeneity of the cell population itself. If there is a need to keep the model simple and preserve reproducibility and feasibility, simplified organoids (spheroids) can be produced starting from a single cell line. Spheroid heterogeneity can be improved stepwise either by co-culturing different cell lines and/or by providing a cocktail of different extracellular matrices. Microfluidic devices are tunable platforms in which cell lines as well as organoids can be cultured. The flexibility of the system allows to compartmentalize and add different components of the tumor including vasculature immune cells and stroma in highly controlled experimental conditions. Given the unique interchangeable geometry of the system, physical stimuli such as stiffness or shear stress can be modulated in the system adding to the level of environmental heterogeneity, known to play a key role in tumor progression. High resolution and live imaging are still the main readouts; thus, good image analysis expertise is required to extract data and succeed in quantifying different parameters including tumor growth and migration. Unfortunately, omics analyses are not always easy to perform given the low number of cells.

Thus, the use of each model should be carefully evaluated in terms of its faithfulness in replicating a given human biological feature or mechanism. In an attempt to define the transcriptional fidelity, the Cancer Genome Atlas dataset has been compared with cell lines, 3D cultures, GEMMs, and PDXs leveraging the CancerCellNet (CNN) tool. Unfortunately, this effort is limited to a small number of tumor-derived models making the validity of this effort very limited.

Recently, immunotherapies have revolutionized the entire cancer treatment field. Immuno-oncology studies and the development of immune checkpoint inhibitors able to boost cancer cell immune recognition have led to recognizing the fundamental role of the immune system in tumor progression. On top of this, increased awareness has been reserved regarding the importance of stromal cells including the ones composing the vasculature and CAFs.

Taking together all this information about the complex hierarchy within the tumor immune microenvironment, it is clear why, despite the different models already available, there is an urgent need to further improve the complexity and fidelity of the platforms replicating humanized settings.

Extending the efforts beyond generating complex models, the need for new technologies to analyze the TME promoted the emergence of single-cell genomics and spatial approaches as powerful strategies in delineating the complex molecular landscapes of cancers.

The acquired knowledge will ultimately be implemented in a digital twin (i.e., “a virtual model” of a physical entity, with dynamic, bi-directional links between the physical entity and its corresponding twin in the digital domain) (268). A digital twin can then be personalized using biological data (269). Personalized digital twins can then be used to test treatment protocols, in the development and identification of new pharmacological targets, and in the rational identification of more effective combined pharmacological protocols that will maximize the therapeutic efficacy for each individual, minimizing the side effects.

In conclusion, understanding tumor heterogeneity and its exploitation in the clinical field will require quantitative determination of multiple features and their integrated analysis by combined machine learning and simulation approaches. Only the combined effort of an interdisciplinary team of scientists with expertise in different fields, such as pathology, molecular biology, bioengineering, clinic, and computation, able to communicate and work in synergy will provide new integration and interrogation modality to predict therapy response and to implement more efficient targeted and combinatorial therapies which are urgently required for cancer patients.

## Author contributions

MG, MV, and MP conceived and designed the review. MG, MV, MP, MC, CD, VP, and ES wrote sections of the manuscript. MG, MP, MC, and CD prepared the figures. MG, MV, and MP reviewed and edited the manuscript. All authors contributed to the article and approved the submitted version.
